# Deep-Learning-Based Automatic Monitoring of Pigs’ Physico-Temporal Activities at Different Greenhouse Gas Concentrations

**DOI:** 10.3390/ani11113089

**Published:** 2021-10-29

**Authors:** Anil Bhujel, Elanchezhian Arulmozhi, Byeong-Eun Moon, Hyeon-Tae Kim

**Affiliations:** 1 Department of Biosystems Engineering, Institute of Smart Farm, Gyeongsang National University, Jinju 52828, Korea; anil.bhujel@gmail.com (A.B.); mohanachezhian@yahoo.com (E.A.); 2 Ministry of Communication and Information Technology, Singha Durbar, Kathmandu 44600, Nepal; 3 Smart Farm Research Center, Gyeongsang National University, Jinju 52828, Korea; be25moon@naver.com

**Keywords:** YOLOv4, Faster R-CNN, Deep-SORT, pig posture detection, object tracking, greenhouse gas, animal welfare

## Abstract

**Simple Summary:**

Animals exhibit their internal and external stimuli through changing behavior. Therefore, people intrinsically used animal physical activities as an indicator to determine their health and welfare status. A deep-learning-based pig posture and locomotion activity detection and tracking algorithm were designed to measure those behavior changes in an experimental pig barn at different greenhouse gas (GHG) levels. The naturally occurring GHGs in the livestock were elevated by closing ventilators for an hour in the morning, during the day, and at nighttime. Additionally, the corresponding pig posture and locomotion activity were measured before, during, and after an hour of treatment. With the increase in GHG concentration, the pigs became less active, increasing their lateral-lying posture duration. In addition, standing, sternal-lying, and walking activities were decreased with the increment in GHG levels. Therefore, monitoring and tracking pigs’ physical behaviors using a simple RGB camera and a deep-learning object detection model, coupled with a real-time tracking algorithm, would effectively monitor the individual pigs’ health and welfare.

**Abstract:**

Pig behavior is an integral part of health and welfare management, as pigs usually reflect their inner emotions through behavior change. The livestock environment plays a key role in pigs’ health and wellbeing. A poor farm environment increases the toxic GHGs, which might deteriorate pigs’ health and welfare. In this study a computer-vision-based automatic monitoring and tracking model was proposed to detect pigs’ short-term physical activities in the compromised environment. The ventilators of the livestock barn were closed for an hour, three times in a day (07:00–08:00, 13:00–14:00, and 20:00–21:00) to create a compromised environment, which increases the GHGs level significantly. The corresponding pig activities were observed before, during, and after an hour of the treatment. Two widely used object detection models (YOLOv4 and Faster R-CNN) were trained and compared their performances in terms of pig localization and posture detection. The YOLOv4, which outperformed the Faster R-CNN model, was coupled with a Deep-SORT tracking algorithm to detect and track the pig activities. The results revealed that the pigs became more inactive with the increase in GHG concentration, reducing their standing and walking activities. Moreover, the pigs shortened their sternal-lying posture, increasing the lateral lying posture duration at higher GHG concentration. The high detection accuracy (mAP: 98.67%) and tracking accuracy (MOTA: 93.86% and MOTP: 82.41%) signify the models’ efficacy in the monitoring and tracking of pigs’ physical activities non-invasively.

## 1. Introduction

Pig behavior is a key trait for recognizing their health and welfare conditions [1]. Regular monitoring of pigs’ physical activity is essential to identify short- and long-term pig stresses [2]. Although the monitoring of pigs round-the-clock in precision farming provides invaluable information regarding their physical and biological status, manual monitoring of every single pig in a large-scale commercial farm is impractical due to the requirement for a higher animal-to-staff ratio, consequently increasing production cost. Therefore, the staff can only observe the pig briefly and might miss identifying subtle changes in the pigs’ activity [3]. Furthermore, the presence of a human in the barn influences the pigs’ behavior, leading to unusual activity that can be misunderstood during the decision-making process [4,5]. Therefore, sensor-based non-disturbing automatic monitoring of pigs is being used considerably.

Numerous studies have shown that the housing environment greatly influences the physical and social behavior of the pigs. Changes in posture and locomotion are key indicators of disease (clinical and subclinical) and compromised welfare [6]. It is also an indicator of pig comfort in the reared environment. For instance, a pig utilizes different lying postures to cope with ambient temperature and maintains body temperature through thermoregulation [7]. They prefer lateral-lying positions in high ambient temperature and sternal-lying at low-temperature conditions. Alameer et al. [8] observed significant changes in posture and locomotion activities with limited feed supply. Identifying subtle changes in pig posture is challenging by sporadic human observation since a pig spends most of the time (88% time in a day) lying in a thermo-comfort environment [7]. Therefore, a computer vision-based automatic monitoring system is valuable, identifying the minute changes in posture through continuous monitoring.

Moreover, there is a burgeoning concern of animal health and welfare in the intensive farmhouse [8,9]. Behavior monitoring is even more pertinent in group-housed pigs as they exhibit significant behavior changes in the compromised environment [10]. Another equally important concern is the emission of greenhouse gases (GHGs) from the extensive livestock farming. Pig manure management is the second-highest contributor (27%) after feed production (60%) in the overall emission of GHGs from the livestock barn. Besides, enteric fermentation and various on-farm energy usage devices produce the major GHGs [11]. Correspondingly, the high GHG concentration inside the livestock barn stems from poor manure management, an improper ventilation system, and densely populated pigs, affecting the pigs’ behaviors [12,13]. Since pigs are averse to an excessive amount of GHGs, such as carbon dioxide (CO_2_), methane (CH_4_), and nitrous oxide (N_2_O), and show discomfort and pain in such environments, it is essential to observe the response of pigs in terms of posture activities with increased indoor GHGs.

CO_2_ and N_2_O, two major GHGs produced in the livestock, are commonly used for euthanizing the pigs, regardless of questions over the pigs’ welfare. Various studies have been conducted by assessing the pigs’ response during stunning. Atkinson et al. [13] observed the changes in pigs’ behavior and meat quality while applying different concentrations of CO_2_. Two different CO_2_ concentrations, 20C2O (20% CO_2_ and 2% O_2_) and 90C (90% CO_2_ in air), were exposed to slaughter pigs, and it was found that pigs felt more uncomfortable in the 90C concentration, with 100% of pigs being stunned. In another experiment, CO_2_, CO_2_ plus Butorphanol, and N_2_O gases were applied to compare stress levels during the euthanization of pigs [12]. Although they were unable to identify the distinction in stress levels in those treatments, N_2_O application could be more humane than CO_2_. Similar results were found by Lindahl et al. [14] in that N_2_O-filled foam could be a suitable alternative to CO_2_ when stunning pigs, improving animal welfare. Verhoeven et al. [15] studied the time taken for the slaughter pig to become unconscious by using different concentrations of CO_2_ (80% and 95%) and studying their corresponding effects on behavior changes. The higher the gas concentration, the quicker the time for the pig to become unconscious (33 ± 7 s). This shows that pigs are significantly affected by a high concentration of GHGs. However, to our knowledge, no study has been conducted to observe the pigs’ behavioral alteration in naturally increased GHGs due to poor livestock management. Therefore, it is essential to monitor the pigs’ behavior in the GHG-concentrated environment, as the livestock barn emits a considerable amount of GHGs [16].

In this scenario, several studies have been conducted for monitoring the pigs’ activity at individual and group levels over the last few decades. The implementation of computer-vision-based monitoring systems in pig barns has been soaring due to the automatic, low cost, real-time monitoring, non-contact, animal friendly, and state-of-the-art performance [17,18,19,20,21,22,23,24,25,26]. An ellipse-fitting and image-processing technique was used to monitor the pigs’ mounting behavior in the commercial farm [18]. Various features such as ellipse-like shape, centroid, axis lengths, and Euclidean distance of head-tail and head-side were extracted to detect the pigs’ mounting position. Similarly, lying behaviors (sternal and lateral lying) at individual and group levels have been classified using image-processing techniques [19,20], where pig bodies from the video frames were extracted using background subtraction and the Otsu threshold algorithm, and then an ellipse-fitting method was applied to determine the lying postures. Matthews et al. [21] implemented a 3D camera and an image-processing algorithm to detect pigs’ behaviors (standing or not standing, feeding, and drinking). The XYZ coordinates obtained from the depth sensor, camera position, and vertical angle of the camera were used to filter out unnecessary scenes such as the floor, walls, and gates. In addition, an outlier threshold calculated from the grand mean and standard deviation was set to remove the unusual depth noise. A region-growing technique for similar pixels was used to detect the pig, whereas a Hungarian algorithm was used to track pigs between the frames. The image-processing technique, although widely used in pig monitoring, demands various pre- and post-processing steps. It is even challenging in an uncontrolled house environment, variable illumination, huddled pigs, and deformed body shapes [17,22].

Accordingly, a convolutional neural network (CNN)-based deep-learning object detection model outperformed the conventional image-processing techniques. Recently, various researches have been carried out using a deep-learning model as an end-to-end activity detection and scoring model rather than only for object detection. A combination of a CNN and a recurrent neural network (RNN) has been used to extract the spatial and temporal features of pigs for tail-biting detection [23], where a pair of bitten and biting pigs from the video frames were detected using a single-shot detector (SSD) with two base networks, Visual Graphic Group-16 (VGG-16) and Residual Network-50 (ResNet-50). A video of tail-biting behavior was sub-divided into a short video of 1 s length to minimize the tracking error. Then, the pairwise interaction of the two pigs was identified by the trajectory of motion in the subsequent frames to detect the biting and bitten pigs. They achieved an accuracy of 89.23% to identify and locate the tail-biting behavior of pigs. Likewise, the pigs’ posture (standing, dog sitting, sternal lying, and lateral lying) and drinking activity were detected automatically using two deep-learning models (YOLO: you only look once; Faster R-CNN) [8]. They found that the YOLO model outperformed the Faster R-CNN (ResNet-50 as a base network) in both activity detection accuracy and speed. They observed the distinction in pig behavior by creating hunger stress and achieved the highest mAP from the YOLO model (0.989 ± 0.009). In addition, the mean squared error (MSE) on the distance traveled by a pig and its average speed were 0.078 and 0.002, respectively.

Similarly, the performance of three deep-learning architectures—namely, Faster R-CNN, SSD, and region-based fully convolutional network (R-FCN) having Inception V2, ResNet, and Inception ResNet V2, respectively, as their base networks—have been evaluated during the detection of pigs’ standing, belly-lying, and lateral-lying activities [24]. The datasets were collected from three commercial pig barns with different colors and age groups of pigs. All the models showed superior detection capabilities (maximum AP of 0.95 compared to standing AP of 0.93 and belly lying AP of 0.92) for the lying by side pigs due to having unique features. Yang et al. [25] developed an FCN-based segmentation and feature extraction model coupled with an SVM classifier to detect sow nursing behavior. Initially, the sow image was segmented from the video frames, and converted into a binary image. Features such as area and length-to-width ratio were extracted to find out the possible nursing conditions. Then, the nursing activity was further confirmed by identifying the udder region using geometrical information from the sows′ shape and the number of piglets present, which was estimated by the area covered by them and their movement. Although this technique required heavy manual effort during the spatial and temporal feature extraction and analysis, it produced state-of-the-art performance on their testing videos (accuracy, sensitivity, and specificity of 97.6%, 90.9%, and 99.2%, respectively).

Even though the deep-learning-based object detection model has surpassed the conventional image-processing technique, due to the limited availability of labeled datasets for wide varieties of piggery environments, it is, therefore, challenging to build a fully generalized model. However, Riekert et al. [26] attempted to develop a generalized deep-learning model using a faster region-based convolutional neural network (R-CNN) with neural architecture search (NAS) as a base network to detect lying or not lying pigs. They applied a large number of training datasets (7277 manually annotated) captured by multiple 2D cameras (20), from various pens (18), prepared from 31 different one-hour videos. The trained model achieved an average precision (AP) of 87.4% and a mean AP (mAP) of 80.2% for the images taken from separate pens with a similar experimental environment during testing. However, the performance reduced significantly (AP of 67.7% and mAP between 44.8% and 58.8%) for those pens with different and complex environmental conditions, which is obvious and signifies that the training dataset is crucial for the deep-learning model to make a generalized model.

Therefore, in this study, a CNN-based deep-learning object detection model, coupled with a real-time tracking algorithm (Deep-SORT), was implemented to detect and track pigs’ standing and lying (sternal and lateral lying) posture along with their locomotion activity in both group-wise and individual. Two commonly used object detection models (YOLOv4 and Faster R-CNN) were trained and compared their performance on pig posture detection. The walking activity of the standing pig was determined by assessing the changes in pig position for the consecutive frames. The distance and speed of the walking pig were calculated by cumulating the movement of the standing pigs within a period. In addition, the GHG concentration in the experimental barn was increased naturally by entrapping the GHGs emitted from the pig barn. The gas samples taken during the study period were analyzed using a gas chromatography. Then, the pigs’ activities during the treatment hours were compared with the activities before and after the treatment hour. Finally, an automatic pig-activity-scoring algorithm was integrated with the trained model for scoring the pig behavior. Thus, the main objective of this experiment was to build a deep-learning-based end-to-end model for the detection and scoring of group-wise and individual pigs’ postures and locomotion activity in the compromised environment.

## 2. Materials and Methods

### 2.1. Experimental House and Animals

This experiment was conducted in an experimental pig barn located at the Gyeongsang National University (Latitude: 35.1517241; Longitude: 128.0958942; and Altitude: 44 M). The pig barn has dimensions of 5.4 m × 3.4 m × 2.9 m (length × breadth × ridge height) with four walls, a symmetrical double-pitched roof of thickness 5 cm, and fully slatted floors [27]. Beneath the slatted floor, there were two boxes for manure collection. The pig house has a feeder system for individual feeding with hydraulic-controlled separate gates for pig entry and exit and three drinking nipples at different heights at the mid-section of a long sidewall. It is equipped with an air damper (Auto-Damper 250, Sanison Co., Ltd., Daegu, Korea) above the door and an exhaust fan opposite the entrance for smooth air movement with an average of 0.16 m^3^/s [28]. Five pigs (four female and one male) of the Yorkshire breed were transferred from the local breeding house in May 2020 and bred up to the fattening stage for experimental purposes. The available floor space for each pig in this experiment was 3.67 m^2^/pig, which was more spacious than the optimum floor space required (0.8 m^2^/pig) in the commercial pig barn in Korea [29]. The pigs were fed twice per day (09:00 and 17:00) with dry feed, and the water supply was continuously available for drinking purposes. The pigs were marked on their backs using different colors and patterns to identify each pig visually, which was taken as a reference while annotating the pig identity for tracking. There was an attached room for monitoring and controlling the system with data collection facilities.

### 2.2. Experimental Setup and Data Collection

**Figure 1:**
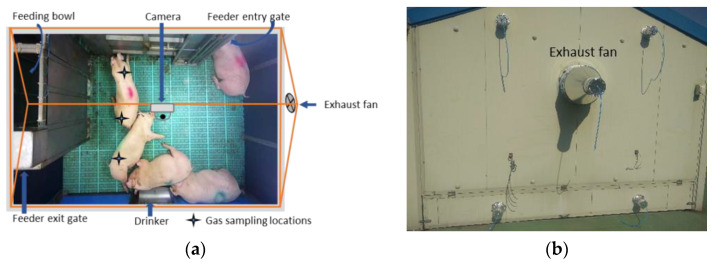
Experimental setup in the pig barn: (**a**) camera setup for capturing the top-view video frames and (**b**) covering of pig barn gas inlet and outlet to elevate the GHGs concentration.

This study was conducted for the fattened pigs (107.14 ± 6.81 kg). The door, ventilator, and damper of the experimental pig barn were closed to increase the GHG concentrations. In addition, the major apertures on the wall, ventilator, and manure boxes were covered, as shown in Figure 1. The treatment was applied for an hour three times a day in the morning time (07:00–08:00), daytime (13:00–14:00), nighttime (20:00–21:00), repeating for three days. During the study period, the average indoor temperature and relative humidity were 26.68 ± 5.23 °C and 57.62 ± 15.14%, respectively. The environmental parameters were maintained and recorded by a livestock environment monitoring system (LEMS) (AgriRobo Tech Co. Ltd., Icheon, Korea). The gas samples were collected before starting treatment, after completing treatment, and one hour later of finishing treatment, using 50 mL syringes [30]. Three gas samples from three different spatial positions were taken at each time, as shown in Figure 1a. Then, the concentration of GHGs was analyzed using gas chromatography (GC) (7890B GC system, Agilent Technologies, Santa Clara, CA, USA). Additionally, the CO_2_ data were also recorded in the LEMS system using two sensors.

A top-view network camera (HIKVISION IP camera, Model: DS-2CD2010-I, HIKVISION Co. Ltd., Hangzhou, China) was installed on the ceiling, with the camera view pointing vertically down. The field of view covered all the pigs that resided on the open floor area, but the pig staying inside the feeder was blocked by the feeder structure. The camera was configured at 30 frames per second (FPS) with a high-definition resolution (1920 × 1080 pixels), which was connected to a network video recorder (NVR) (HIKVISION 4K NVR, Model: DS-7608NI-I2/8P, HIKVISION Co. Ltd., Hangzhou, China) to store the video throughout the experiment period. The camera was also accessed through its application programming interface (API) [31] in Python and saved every second frame in the server for the training and testing purpose of the model. However, the pigs’ activities during the study period were analyzed from the video files exported from the NVR. In each experiment instance, three hours of videos (before, during, and after the treatment) were analyzed. Therefore, throughout the study period, a total of 27 h of videos (3 × 3 × 3 h) were examined.

### 2.3. Image Pre-Processing and Dataset Preparation

The raw images were pre-processed before further utilization. The original image of dimensions 1920 × 1080 was resized to 640 × 640 pixels to meet the requirements of the pre-trained deep-learning model. Moreover, the pig postures (sternal lying, lateral lying, and standing) were localized and labeled on the resized frames. Before annotation, the similarity between the consecutive frames was checked because, in every second frame, the adjacent image frames were not varying significantly, as the pigs mostly remained inactive. We used a perceptive hash (pHash) technique [32], which generates 64-bit long hash values according to the visual appearances. This was to provide diverse training images, increasing the robustness of the model while testing. A discrete cosine transform (DCT) of an image was calculated, and 8 × 8 transform coefficients were selected and raveled to make a 64-bit one-dimensional array. A median was calculated from the 64 DCT coefficients and then 64-bit long hash values were created using Equation (1):

(1) $$h_{i}=\{\begin{array}{c} 0,C_{i}<m\\ 1,C_{i}\geq m \end{array}$$

where $h_{i}$ is an *i*th position bit of the pHash value, $C_{i}$ is the *i*th position’s DCT coefficient, and m is the median of DCT coefficients. The hash distance was set to 1 to filter out only the too similar frames. In addition, the corrupt frames that occurred during the image collection were removed. In this way, a total of 6680 pre-processed images were prepared and annotated manually using a computer vision annotation tool (CVAT) [33]. Some of the guidelines set by the Pascal VOC 2010 [34] were followed while annotating the pig postures. As per the guidelines, if more than 15–20% of the object is not covered by the bounding box (BB), it needs to be marked as truncated and a BB drawn to cover only the visible part. Similarly, for the occluded object, the BB is drawn to cover all the visible parts, setting the occluded flag if the occlusion is more than 5%. We followed the BB generation method similar to the guidelines except the setting of the truncated and occlusion flag [26].

Although there are no standard datasets available for pig posture, three posture categories of pigs (standing, lateral lying, and sternal lying) were labeled manually in this study according to the convention applied by Nasirahmadi et al. [19] and Alameer et al. [8], as shown in Table 1. The annotated information was saved in Pascal VOC format and later converted into TFRecord format for the Faster R-CNN model and in Darknet format for the YOLO model. The total number of annotated postures was 30,233. The labeled images were then randomly split into training and testing datasets at a ratio of 90:10. Thus, 6012 images were used for model training, and the remaining (668) images were used for testing purposes. We had opted to select the 90:10 ratio for training and testing because of the limited number of labeled datasets. The trained model was later utilized for detecting the pigs’ postures in the images collected during the study periods. The labeled testing datasets are made available in the supplementary files [Datasets].

For the tracking dataset, one-minute video clips after downsizing to 5 frames per second (FPS) were annotated in a similar way to the pig detection dataset with an additional pig ID assigned to each pig. All the pigs were marked by a color marker with a distinct pattern, which helped to set the pig IDs even in different video clips. If a pig was out of a frame and later visible into the frame, we gave the same ID to that pig.

### 2.4. Proposed Methodology

**Figure 2:**
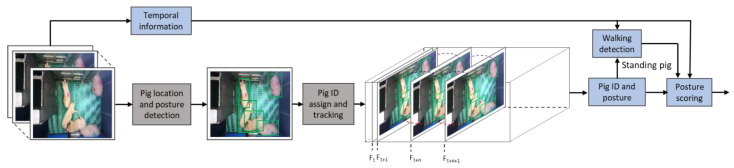
Complete steps of proposed methodology. The pig postures are identified and localized by using a deep-learning object detection model, then the simple online real-time tracking with deep association (Deep-SORT) algorithm tracks each pig by associating the corresponding detected posture, and, finally, the detected pigs’ postures with time information are analyzed to score the posture activity occurring in an hour. All the modules are integrated to provide an end-to-end posture scoring.

The prime objectives of this study were to detect the pig postures in a frame, tracking them individually, and finally scoring each pig posture with time. Therefore, the foremost task in the input video frame was to detect the pig posture and localize it in the given frame. Secondly, a tracking algorithm associated the detection metric with an ID and maintained the same ID for the successive frames. Finally, the quantification of pig activities during the study period was performed. The complete steps of the proposed methodology are shown in Figure 2. The posture detection model and tracking model were trained separately. Then the trained models were coupled together with a posture scoring algorithm to make a complete end-to-end pig posture scoring model.

#### 2.4.1. Pig Posture Activity Detection Model

**Figure 3:**
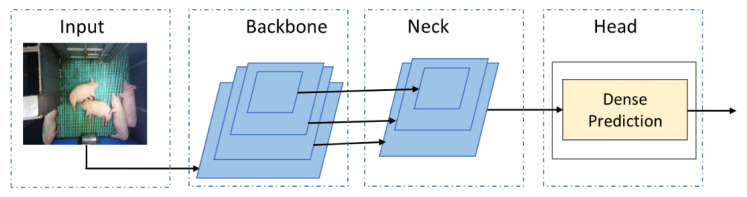
YOLOv4 network architecture.

YOLOv4 model: YOLO is a prominent object detection model that outperforms other detection models in terms of both accuracy and speed. Moreover, the latest version of the YOLO series improved significantly in both object detection accuracy and speed. This study uses YOLO version 4 [35] due to its superior performance than the earlier versions in public datasets. It works as a single-stage object detection model, which speeds up the object detection time. The network architecture of YOLOv4 consists of three blocks, as shown in Figure 3. The backbone block uses the deep convolutional neural network CSPDarknet53 (cross-stage partial connections Darknet53) for feature extraction. The neck block uses a spatial pyramid pooling (SPP) and path aggregation network (PAN) to concatenate and fuse the different-sized feature maps. The head block uses the dense prediction network implemented by YOLOv3 [36] to predict the bounding box and class. The significant improvement in the methodological, regularization, and data augmentation techniques placed the YOLOv4 on top.

**Figure 4:**
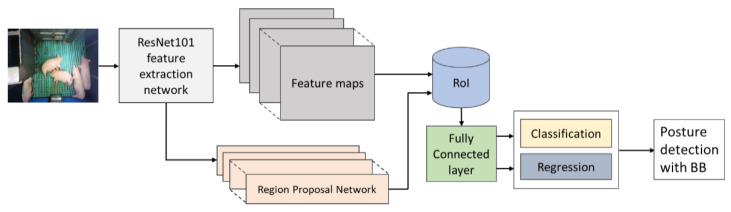
Architecture of Faster R-CNN.

Faster R-CNN model: A pre-trained object detection model trained by a large number of Microsoft common object in context (COCO) datasets [37] was downloaded from a GitHub archive [38] and re-trained to detect pig posture. A Faster R-CNN [39] with ResNet101 with the input image size of 640 × 640 as a feature extractor was implemented (Figure 4). It consisted of two modules, a region proposal network (RPN) and a Fast R-CNN detector [40]. First, an RPN module, a fully convolutional network that generates region proposals, was implemented. In RPNs, an *n* × *n* spatial window slides over the feature maps generated from the last shared convolutional layer to generate bounding boxes. The RPN shared the image features extracted by the object detection network resulting in faster computation than Fast R-CNN. It predicts the region proposal using anchor boxes of different sizes and aspect ratios to speed up the training and testing process. Second, a Fast R-CNN detector, which uses the proposed regions provided by the RPN, was implemented. A Faster R-CNN object detection model was chosen as it produces a satisfying average precision on pig posture detection [3,23].

#### 2.4.2. Pig-Tracking Algorithm

**Figure 5:**
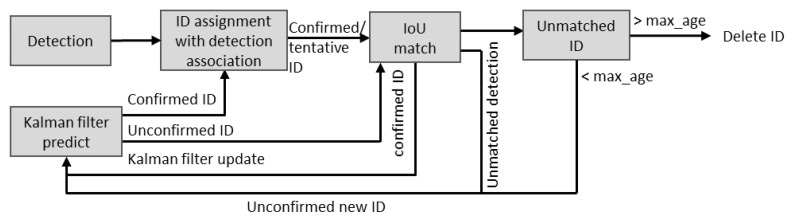
Pig-tracking algorithm adopted in Deep-SORT. A virtual ID is assigned to each detected pig in the first frame, and the IDs are given to the respective pigs based on the matching IoU in the subsequent frames.

After detecting and localizing the pig postures in a frame, a visual- and distance-based tracking algorithm called deep simple online real-time tracking (Deep-SORT) [41] was implemented to track the pigs individually. The Hungarian algorithm [42], implemented alongside, preserved the individual pig identity in consecutive frames, allowing for the detection of a lost or new pig in the next frame. The location of the missed pig was estimated using the Kalman filter [43]. Thus, it can track the pigs even in certain occlusions and frame corruptions. However, errors in detection led to deterioration of the tracking performance. Moreover, the pig posture obtained from the detection model was associated with an ID to determine the individual pig’s profile. The overall flow diagram of the pig-tracking algorithm is presented in Figure 5 [41]. In this study, we used offline video clips for analysis. Therefore, all the video files were sub-divided into 1-min video clips. Then the IDs of pigs in the first frame of each video clip were assigned based on the hierarchy of the detected pigs, allowing the algorithm to track for a minute.

#### 2.4.3. Pig-Moving Detection and Activity-Scoring Algorithm

When the center coordinates of the standing pig changed more than the threshold pixels (4 pixels), the standing pig was considered as a walking pig [3]. The Euclidean distance (∆d) between the centroid of the standing pig in the consecutive frames was calculated to check the movement, and the total distance covered by the pig was obtained by summing the ∆ds for an hour. Likewise, the average speed of the moving pig was determined by dividing the total distance covered by the time taken. Moreover, the posture scores of the pigs at the individual and group levels were obtained using Equation (2) [8]:

(2) $$P_{i}\left(a\right)=\frac{\sum_{k=1}^{n}AF_{k}}{n}$$

where $P_{i}\left(a\right)$ is the posture activity (for instance, lateral lying) of an *i*th pig, $AF_{k}$ is the *k*th frame that has the ith pig’s *a*th activity, and *n* is the total number of frames in a video clip. We first identified the individual pigs’ posture within one-minute video clips and then integrated them hour-wise to see the changes in behavior before, during, and after an hour of the treatment.

#### 2.4.4. Training and Evaluation of the Model

The selection of a suitable learning rate in the deep-learning model is crucial. However, there is no ready-made solution for selecting the best learning rate in machine learning. The higher the learning rate, the quicker the learning speed and vice versa. However, too high and too low learning rates will not converge the network effectively [44]. As per the number of training datasets and the network structure, the training hyperparameters were chosen, as shown in Table 2 [35] and Table 3 [24].

The evaluation of the trained model was carried out on validation datasets. The intersection over union (*IoU*), which measures how accurately the model creates a bounding box of a pig in the frame, was calculated using an Equation (3) with a threshold of 0.6 [3]. The model’s localization performance is good when the detected bounding box and ground-truth bounding boxes have an *IoU* close to 1.

(3) $$IoU=\frac{BB_{GT}\cap BB_{P}}{BB_{GT}\cup BB_{P}}$$

where $BB_{GT}$ is a ground-truth bounding box, $BB_{P}$ is a predicted bounding box, ∩ is an intersection operator that calculates the common area covered by $BB_{GT}$ and $BB_{P}$, and ∪ is a union operator that obtains the total area covered by both $BB_{GT}$ and $BB_{P}$. Moreover, mean average precision (mAP), a widely used metric in object detection, was used to evaluate the model’s detection performance.

For the tracking algorithm, the commonly used metrics mentioned in the MOT 2016 MOT Challenge [45] were used. Multi-object tracking accuracy (*MOTA*) and multi-object tracking precision (*MOTP*), as given in Equations (4) and (5), respectively, were used to evaluate the tracking algorithm:

(4) $$MOTA=1-\frac{\sum_{i}(FN_{i}+FP_{i}+IDSW_{i})}{\sum_{i}GT_{i}}$$

where $FN_{i}$ is the false negative (untracked pigs in the *i*th frame), $FP_{i}$ is the false positives (wrongly tracked the pigs in the *i*th frame), $IDSW_{i}$ is the identity-switched pigs (given a new *ID* in the *i*th frame for the same pig in the previous frame), and $GT_{i}$ is the ground truth of pigs.

(5) $$MOTP=\frac{\sum_{t,i}d_{i}^{t}}{\sum_{i}c_{i}}$$

where $d_{i}^{t}$ is the distance or *IoU* between the ground-truth bounding box of an object and target t in the *i*th frame, and $c_{i}$ is the number of ground-truth targets in the *i*th frame.

The training and evaluation of the model were performed in Python 3.7.10 installed on the Windows 10 Pro operating system. The hardware configurations of the computer were Intel Core 10th generation i9-10900k processor with 32 × 2 GB RAM and an NVIDIA GeForce RTX 2070 GPU with 8 GB of dedicated memory. The main codes and algorithms are provided in the supplementary files [Code].

## 3. Results

### 3.1. Greenhouse Gas Concentrations

**Figure 6:**
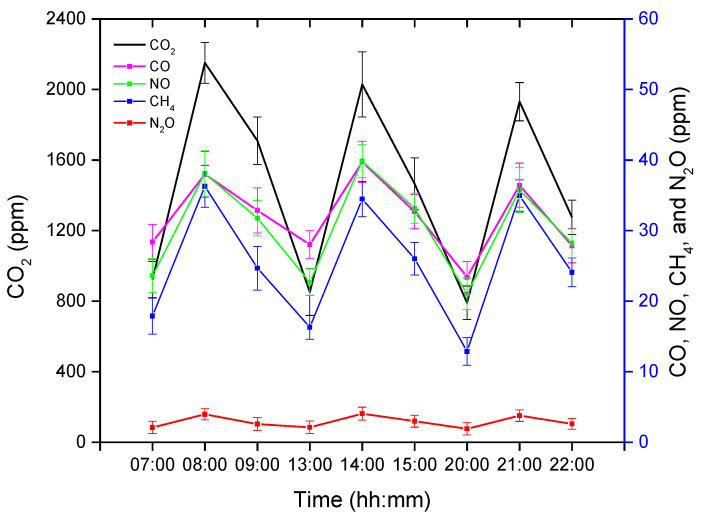
Average greenhouse gas (GHG) concentrations before, during, and after an hour of treatment. The x-axis represents the time (hh:mm) of day, whereas the left y-axis represents the average CO_2_ concentration, and the right y-axis represents the average CO, NO, CH_4_, and N_2_O.

The GHG concentrations obtained after analyzing the gas samples were averaged and are presented in Figure 5. The GC has the ability to detect five varieties of GHGs, namely carbon dioxide (CO_2_), methane (CH_4_), carbon monoxide (CO), nitric oxide (NO), and nitrous oxide (N_2_O). CO_2_ is the dominant GHG, followed by CO and NO, whereas N_2_O was found in the lowest concentration in this experimental pig barn. The GHG concentrations were measured three times in each treatment instance (before, after, and one hour later of treatment completion), as shown in Figure 6.

### 3.2. Group-Wise Pig Posture and Walking Behavior Score

**Figure 7:**
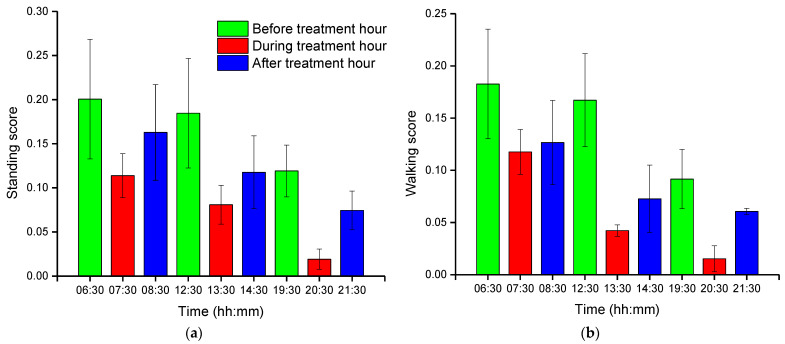

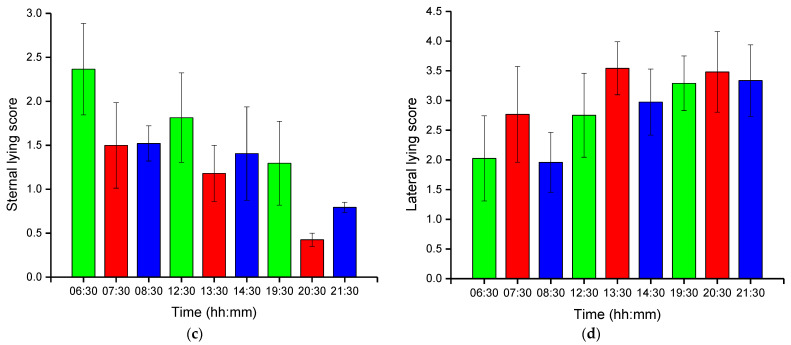
Group-wise average posture and walking frame scores of pigs: (**a**) standing score, (**b**) walking score, (**c**) sternal-lying score, and (**d**) lateral-lying score. The scores show the average number of pigs in a frame with a particular posture. The bars represent the average activity scores obtained in three days at an hour before, during, and after the treatment period in the morning, day, and nighttime.

**Figure 8:**
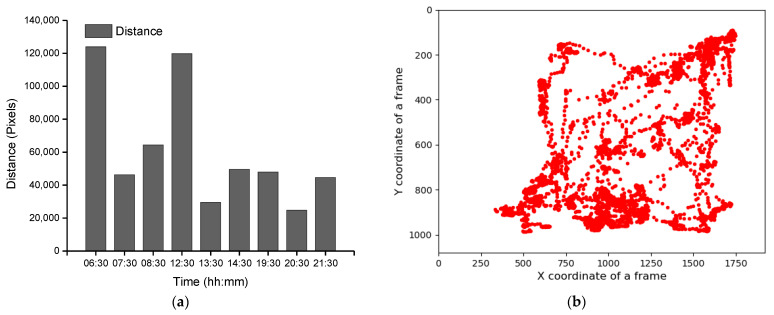

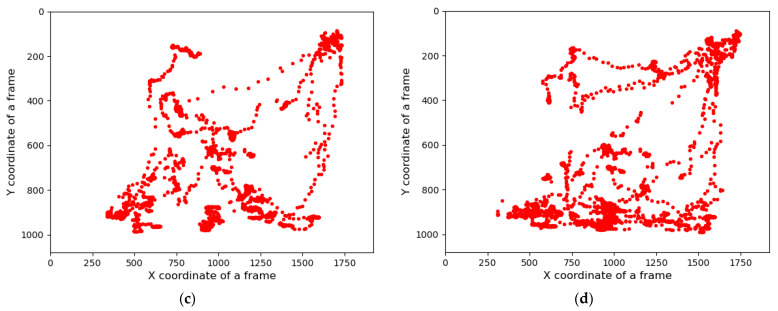
Total distance traveled by the pigs in group and the day 1-morning time locomotion activities: (**a**) the total distance traveled by all pigs during the study period, (**b**) locomotion of the group pigs before treatment in the morning of day 1, (**c**) during the treatment, and (**d**) after the treatment.

Group-wise pig posture and walking activity scores are measured by dividing the number of pigs with a particular posture by the number of frames before, during, and after treatment hour. Most of the time, pigs stayed in the lying position (sternal and lateral). However, the pigs were more active in the morning compared to the day and nighttime. Moreover, at night, pigs primarily rested in the lateral lying position. With the peak value in GHGs, the standing and walking activities of the pigs were decreased significantly (almost by half), as shown in Figure 7a,b. The standing score was increased with the decrease in GHGs (one hour later of treatment). A similar pattern was followed by the walking activity score, except in the morning, where the walking score did not increase noticeably. Likewise, the sternal lying behavior of the pigs also decreased with the increase in GHGs (Figure 7c). Conversely, the lateral lying behavior of the pigs increased significantly in the morning and daytime (nearly 40% in the morning and 30% in the day). However, it was marginally increased in the nighttime, as given in Figure 7d. One hour later of the treatment (08:00–09:00, 14:00–15:00, and 21:00–22:00), the GHGs remained relatively higher than before the treatment. Therefore, the respective effects on all the pigs’ activities were observed, as presented in Figure 7. The total distance traveled (in terms of pixels) by all walking pigs was higher in the morning before treatment hour and observed least in the nighttime treatment hour. Figure 8 demonstrates the total distance traveled and the locomotion pattern of walking pigs on the morning of the day 1 experiment. The detected pig postures with the locomotion activity and pig identifications are available in the supplementary file [Result].

### 3.3. Individual Pig Posture and Walking Behavior

**Figure 9:**
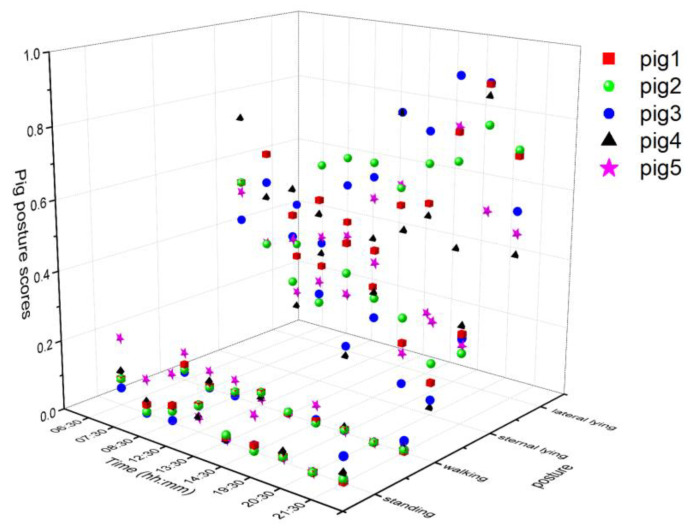
Posture and walking activity score profiles of individual pigs during the study period.

The tracking algorithm provided a virtual ID to each pig and hept tracking them until the number of missing frames was less than the specified age of the ID (50). The individual posture and walking activities were determined similarly as group-wise behavior measurements, except treating them individually. The posture and walking activity scores of each pig are given in Figure 9. Pig 5 was more active than the other pigs, whereas Pig 3 was inactive most of the time.

### 3.4. Pig-Activity Detection and Tracking Model Performance

**Figure 10:**
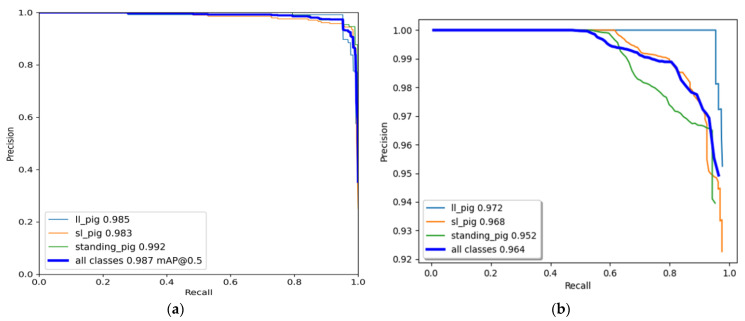
Class-wise and overall precision-recall evaluation metrics of the model: (**a**) YOLOv4 model and (**b**) Faster R-CNN model.

**Figure 11:**
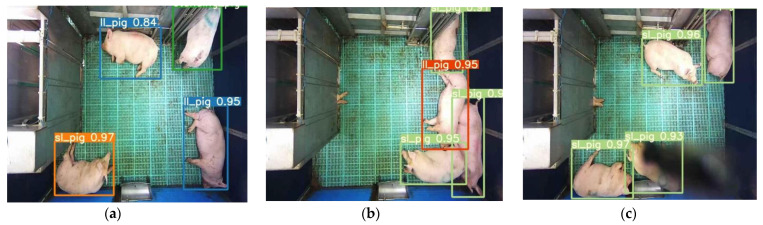

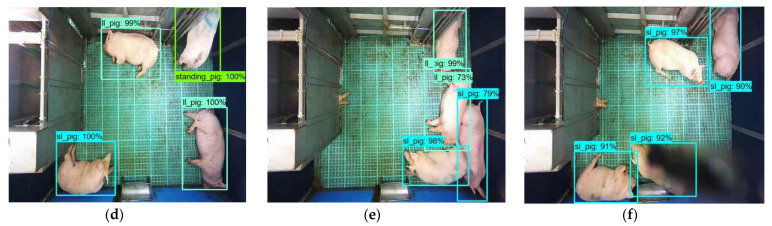
Sample of pig posture detection under various conditions by two models: (**a**–**c**) by YOLOv4 model for the frame having sparsely located pigs, frame having densely located pigs, and the frame having occlusion, respectively, and (**d**–**f**) by Faster R-CNN model for the same frames applied in the YOLOv4 model.

The YOLO model was trained for 500 epochs, whereas the Faster R-CNN model was trained for 50,000 iterations and saved with trained weights. Then, the model performance was assessed comprehensively using a toolkit implemented in the object detection metrics analysis [46]. The overall and class-wise average precision and recall obtained from the two models are shown in Figure 10. The YOLO model gave balance accuracy metrics (Figure 10a) compared to the Faster R-CNN model, which produced the highest accuracies for lateral lying posture detection and least for standing posture (Figure 10b). The APs of the Faster R-CNN model for the lateral lying, sternal lying, and standing postures were 97.21%, 96.83%, and 95.23%, respectively, with an overall mAP of 96.42% at 0.5 IoU. In comparison, the YOLO model provided 98.52%, 98.33%, and 99.18% accuracies for lateral lying, sternal lying, and standing postures, respectively, with an overall mAP of 98.67% at 0.5 IoU. Figure 11 shows the example frames of pig posture detection in different scenarios. The detection confidence of the Faster R-CNN model was higher for the sparsely located pigs. However, it declines with the increase in pig congestion and occlusion occurrence. Whereas the YOLO model produced balanced detection confidence in all scenarios, providing better precision and recall values. The Faster R-CNN model provided some false positive detections for standing and sternal lying postures, reducing the precision score, as shown in Figure 10b. The posture dections by the models for a sample video is provided as supplementary files. Similarly, the time taken by the YOLO model was 0.0314 s per image compared to 0.15 s per image of the Faster R-CNN model. The models’ detection speed was calculated by averaging the time taken to detect 30 min video frames.

**Figure 12:**
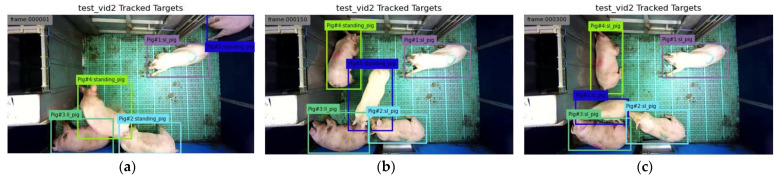
Sample of tracked frames by the tracking algorithm: (**a**) the first frame, (**b**) the 150th frame as Pig 5 is moving, and (**c**) the 300th frame where Pig 5 has changed its posture from standing to sternal lying.

Our proposed model works on tracking by detection strategy. The pig location in the frame was detected by the YOLO model with the corresponding pig posture. Then, the tracking algorithm assigned a virtual ID to the detected pig and tracked it throughout the frames in a video clip. Therefore, the accuracy of the tracking algorithm also depends upon the accuracy of the detection algorithm. In this study, the YOLOv4 produced good detection accuracy (98.67%), resulting in a good tracking accuracy of MOTA 93.13% and MOTP 81.23%. Some example frames after implementing the tracking algorithm are shown in Figure 12. Sample videos of pig posture detection from the YOLO model [Video S1], from the Faster R-CNN model [Video S2], and the tracking algorithm [Video S3] have been provided as supplementary files.

## 4. Discussion

**Figure 13:**
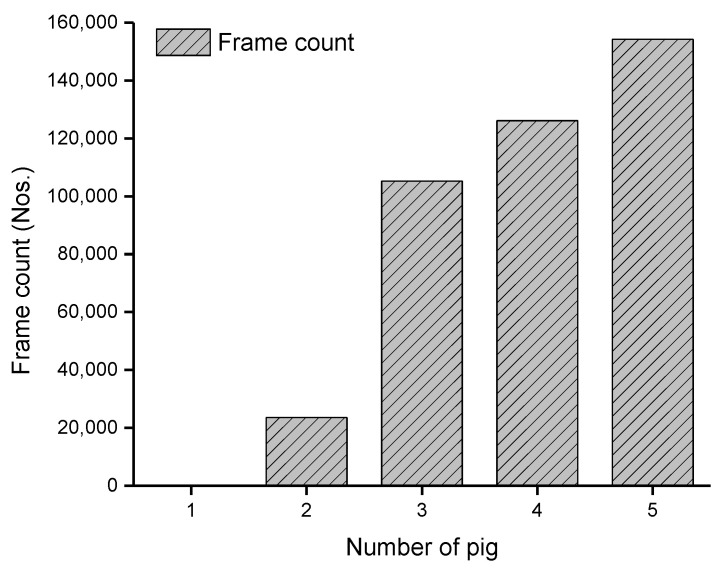
Number of frames vs. number of visible pigs during the study period.

Although the pig posture detection accuracy of the model is impressive, this study has some limitations. In this section, we discuss those limitations with the inference of the results. The first limitation is the pen environment. A big-sized feeder structure obstructed capturing all the pigs throughout the study period, especially those who stayed inside the feeder. Figure 13 shows the number of frames with the different numbers of visible pigs. Not all pigs were visible throughout the study period; nonetheless, the majority of pigs were visible in most of the video frames. This might be the one reason for getting a higher standard deviation while analyzing the group-wise activity (Figure 7). The second limitation was the manual system for gas sample collection and treatment application, which disturbed the natural activity of pigs for a while. Therefore, the video frames (10 min after the human entrance) were not considered for the activity analysis. In addition, the treatment duration (1 h) was set empirically. However, further research is recommended to identify the minimum duration and GHG concentration required for the pig to feel discomfort.

Even though there was no spatial variation in the GHGs in the livestock barn [47], we took three air samples from three spatial locations near the center of the pig barn each time and averaged them, as shown in Figure 1. The acquisition of GHG values using the air sampling method collects the air samples near the center of the barn. Dong et al. [47] collected air samples from three pen sections near the middle area of each pen. The space of two pens had an equal size of 4 m × 7 m each, and the third one had 8 m × 8 m. Likewise, Ni et al. [48] chose the one-one location for each pen near the exhaust fan (1 m from central exhaust fan) and one at the center of the aisle in the large pig barn of size 61.0 m × 13.2 m. Compared to those pig barns, our pig barn was small in size (5.4 m × 3.4 m), and we have collected three gas samples from three different locations and averaged them so that the GHG values would be more reliable. Dong et al. [47] reported that the GHG concentration profile in the pig barn has diurnal variations. A similar pattern was observed in our pig barn, with the concentration being higher in the nighttime and lower in the daytime. All the GHG concentrations displayed almost a similar pattern. The GHG concentrations in the morning were high because of the reduced ventilation at low temperatures during the nighttime, resulting in highly concentrated GHGs. CO_2_ gas was found in considerably higher concentrations than other GHGs. Previous research has also reported that CO_2_ and CH_4_ are the major GHGs produced in the pig barn, and their concentrations are highly correlated with farm management systems [47,48]. CO_2_ mainly occurs from pig respiration, with some from manure, and CH_4_ comes from the fermentation of pig waste. Our pig barn was equipped with a shallow manure-flushing system, which was flushed every week, which might be the reason for there being less CH_4_ concentration compared to other GHGs, as Moller et al. [49] found that the CH_4_ emissions from pig waste is dependent on the storage time. The GHGs were increased by more than 100%, with the turning off of the ventilation system for an hour. Thus, poor farm handling may produce a severe level of GHGs inside the pig barn causing stress on the pig.

CO_2_ is a commonly used GHG for stunning the pigs before slaughter. Recently, some research on N_2_O and N_2_ gases has been carried out as a substitute or a supplementary of CO_2_. However, CO_2_ and N_2_O with various other hazardous gases are produced naturally in the animal house, and the concentration of emissions largely depends on the farm management practices. We have developed a model to identify the pigs’ response to elevated GHG concentrations. Verhoeven et al. [15] reported that the increase in CO_2_ concentration decreases the sitting and lying latency, increasing their durations. Nevertheless, there was no significant difference in walking activity. Similar behavioral responses were observed in this study, except for walking activity, which might be due to the difference in CO_2_ concentration and exposure method. There is still a debate on the amount of CO_2_ concentrations required to create aversion to pig since it responds differently in different studies. Nevertheless, more than 30% of CO_2_ inhalation by volume in air and 15 to 30% of CO_2_ in nitrogen atmosphere causes aversion to pig [50]. In the case of humans, CO_2_ of more than 1000 ppm shows disturbance on cognitive performances, and above 3000 ppm significantly increases headache, sleepiness, and exhaustion [51]. When a higher amount of CO_2_ is inhaled, it spreads on the blood, reducing the pH level since it is mildly acidic, resulting in a state of hypercapnic hypoxia. Therefore, pigs became more inactive at higher concentrations of GHGs. Also, the CO_2_ is a strong respiratory stimulator, showing the gasping and hyperventilation activities on the pig [15]. A pig ethogram is an essential parameter for monitoring its health and welfare. Alameer et al. [8] found a decrease in standing activity in response to a certain level of food stress, which increased sharply when the stress increased. However, the lateral lying activity increased sharply even under initial food stress and decreased gradually with the increase in food stress, but overall, it was higher in stress conditions. However, the sternal lying activity changed remarkably with severe food stress only. The pigs showed similar behavior in the GHG stress as well. The standing, walking, and sternal lying decreased with the increase in GHGs in contrast to lying laterally for a longer time. Moreover, with the increase in GHGs, the pigs might feel difficulty in breathing, causing stress on the pigs. Stress on the pigs also increases their body temperature [52], and hence the pigs are likely to stay in the lateral-lying position [53]. Therefore, monitoring the pigs’ ethogram using a camera would be pertinent in identifying the pigs’ welfare. Furthermore, the individual monitoring and tracking of the pig will provide essential information for identifying each pig’s health and welfare conditions. For instance, Pig number 5 was found the most active pig, and pig number 3 was the most inactive.

As we used an automatic posture detection and tracking model to observe changes in the pigs’ behavior, the reliability of the posture observation is inevitably dependent on model performance. Our model produced an excellent detection result (mAP: 98.67%), which is better than [3,24] and similar to [8]. The higher detection accuracy might be due to the latest model, lower pig population, clean pen environment, enough lighting, single pig breed, and clear and large pig body. Similar to [8,24], the AP of the Faster R-CNN model for the lateral-lying class was the highest among the postures, whereas the AP for the standing class was the lowest, as shown in Figure 8b. However, the YOLO model provided balanced APs for all classes of pig posture (relatively higher for standing posture and the lowest for sternal lying posture). Implementation of an SPP network in the neck block improved the detection accuracy of the model, even for some deformed object shapes. For the top-view images, sometimes, the standing pig looked similar to a sternal-lying pig [24]; thus, the models got higher false positives and false negatives, reducing the APs of those classes. However, the YOLO model showed a robust performance on all posture detections. Additionally, models’ classification and localization confidence decreased in huddled and posture transitional scenes, which should be improved in the future. However, the model performed satisfactorily in all the posture classes, which is inspiring for its implementation in pig posture detection. To our knowledge, this is the first study on pig behavior measurement using deep-learning networks under various GHG concentrations. Also, this study focuses only on the short-term effect of GHG concentrations on pig behavior. Therefore, further research is recommended to identify the long-term impact on pig growth performance.

## 5. Conclusions

The posture and locomotion activity of pigs is a vital indicator for health and the monitoring of well-being. We developed a deep-learning-based automatic algorithm utilizing the computer vision system for measuring the changes in such behaviors under a compromised breeding environment. Two widely used object detection models (YOLOv4 and Faster R-CNN) were implemented and adopted the YOLO model due to its fast and accurate detection. We observed the changes in pig posture both group-wise and individually at the different concentrations of GHGs that occurred naturally in an experimental pig barn. The detection model performed remarkably with mAP of 96.42%, and the tracking algorithm provided a MOTA and MOTP of 93.13% and 81.23%, respectively. The tracking algorithm allowed to find out the individual pig-activity profiles during the study period.

Even though we did not find previous research regarding the influence of GHG concentrations on pig behavior changes, our study showed significant differences in pig posture activities. Standing, walking, and sternal lying activities were inversely correlated with the GHG concentrations, whereas lateral lying showed a positive correlation. However, there needs to be further experiments to determine the impact of GHGs on pigs’ health and growth.

## Figures and Tables

**Table 1:** Pig posture categories and their convention used to classify them in human annotation.

Pig Posture and Label	Identification Convention	Instances
Standing pig (standing_pig)	Only feet or feet and snout in contact with the floor	10,124
Sternal lying pig (sl_pig)	Belly and folded limbs in contact with the floor	9364
Lateral lying pig (ll_pig)	Side trunk and extended limbs in contact with the ground	10,745

**Table 2:** Hyperparameters selected for the training of the YOLOv4 model.

Hyperparameter	Value
Learning rate	0.001
Epochs	500
Optimizer	Adam
Batch size	2
Subdivisions	1
Activation	Mish
Input image size	[640, 640, 3]
Data augmentation	Horizontal and vertical flip, Rotations by 90°, 180°, and 270°, and mosaic augmentation

**Table 3:** Hyperparameters selected for the training of the Faster R-CNN model.

Hyperparameter	Value
Learning rate	0.004
Iteration	50,000
Warmup learning rate	0.0013333
Momentum	0.9
Batch size	2
Score converter	Softmax
Input image size	[640, 640, 3]
Data augmentation	Horizontal and vertical flip; Rotations by 90°, 180°, and 270°

## Data Availability

The code, algorithm, and testing datasets are provided as Supplementary Materials.

## Supplementary Materials

The following are available online at https://drive.google.com/file/d/1DmkR5AyysQkFbMEwjPjJnnNVyGvtsu9H/view, Video S1. Sample video of pig posture detection from the YOLO model; Video S2. Sample video of pig posture detection from the Faster R-CNN model; Video S3. Sample video of pig tracking; Code. Pig tracking by detection algorithm code; Datasets. Labeled testing datasets; Result: Pig posture detected and tracking results.
